# Aspirin use and cancers of the upper aerodigestive tract

**DOI:** 10.1038/sj.bjc.6600820

**Published:** 2003-03-04

**Authors:** C Bosetti, R Talamini, S Franceschi, E Negri, W Garavello, C La Vecchia

**Affiliations:** 1 Istituto di Ricerche Farmacologiche ‘Mario Negri’, Via Eritrea 62, 20157 Milan, Italy; 2 Servizio di Epidemiologia, Centro di Riferimento Oncologico, Via Pedemontana Occ.le, 33081 Aviano (Pordenone), Italy; 3 International Agency for Research on Cancer, 150 Cours Albert Thomas, F-69372 Lyon Cédex 08, France; 4 Ospedale ‘San Gerardo’, Università di Milano Bicocca, Via Donizetti 106, 20052 Monza, Italy; 5 Istituto di Statistica Medica e Biometria, Università degli Studi di Milano, Via Venezian 1, 20133 Milan, Italy

**Keywords:** upper aerodigestive tract cancer, aspirin, case–control study, risk factor

## Abstract

The role of aspirin on the risk of cancers of the upper aerodigestive tract was investigated in the combined data of three Italian case–control studies, including 965 cases and 1779 hospital controls. The odds ratio was 0.33 for users of ⩾5 years, and 0.51 for ⩾5 years since first use.

Data on the role of aspirin on the risk of cancers of the upper aerodigestive tract are scanty, and mainly related to cancer of the oesophagus. In two rheumatoid arthritis cohorts from Finland (Isomäki *et al*, 1978) and Sweden (Gridley *et al*, 1993), no significant association between aspirin use and oesophageal cancer risk was observed (relative risk (RR)=0.94 and 1.3, respectively). In a large prospective study from the American Cancer Society, a significantly reduced risk of oesophageal cancer (RR=0.59) was reported in subjects who used aspirin ⩾16 times per month for at least 1 year, and the risk reduction was stronger among users for ⩾10 years (RR=0.54) (Thun *et al*, 1993). In the same study, a nonsignificant increased risk was observed for oral and pharyngeal cancer. In the cohort of the National Health and Nutrition Examination Survey, aspirin use was associated with a 90% decreased risk of developing oesophageal cancer, although the estimate was based on 15 cases only (Funkhouser and Sharp, 1995). A US population-based case–control study, including 221 squamous-cell oesophageal cancer and 293 adenocarcinoma cases, found a significant lower risk for current aspirin users (odds ratio (OR)=0.49 for squamous-cell and 0.37 for adenocarcinoma) (Farrow *et al*, 1998). In a British case–control study conducted on 159 women with squamous-cell oesophageal cancer, 10 cases *vs* 19 controls reported taking aspirin daily for at least 1 month (crude OR ⩾0.50) (Sharp *et al*, 2001). In a case–control study from Greece with 43 squamous-cell cancers and 56 adenocarcinomas of the oesophagus, the OR was nonsignificantly reduced for ever aspirin use (Garidou *et al*, 1996).

The potential role of aspirin use on cancers of the upper aerodigestive tract was further investigated using data from three case–control studies conducted in Italy.

## PATIENTS AND METHODS

Three hospital-based case–control studies of cancer on the upper aerodigestive tract were conducted in Italy between 1992 and 2000 to examine alcohol, tobacco, diet, family history and other environmental factors, covering a total of 1362 cases and 3038 hospital controls (Franceschi *et al*, 1999; Bosetti *et al*, 2000; Talamini *et al*, 2002).

Controls were from the same hospitals as cases, admitted for a wide spectrum of acute, non-neoplastic conditions, not related to smoking, alcohol consumption or long-term modifications of diet; they were frequency-matched with cases by 5-year age groups, sex and study centre. To compensate for the rarity of laryngeal cancer in women, a control-to-case ratio of about 5 was chosen for women, as opposed to 2 for men. On average, 5% of cases and controls approached during their hospital stay refused to be interviewed (Franceschi *et al*, 1999; Bosetti *et al*, 2000; Talamini *et al*, 2002).

The interview-administered questionnaire included information on sociodemographic characteristics, anthropometric measures, lifestyle habits, including tobacco smoking and alcohol drinking, a validated food frequency section and personal and family medical history. The information on aspirin intake included indication, age at first use, frequency and duration of use. Regular use was defined as use for at least once a week for more than 6 months. To facilitate recall of use, a comprehensive list of major aspirin-containing preparations in Italy was supplied.

A total of 965 cases (850 men, 115 women) of the upper aerodigestive tract cancer (393 oral and pharyngeal, 225 oesophageal and 347 laryngeal cancer) and 1779 (1450 men, 329 women) controls with information on aspirin use were included in the present analysis.

ORs and the corresponding 95% confidence intervals (CI) were estimated using unconditional multiple logistic regression models (Breslow and Day, 1980), including terms for age, sex, study centre, years of education (<7, 7–11, ⩾12), alcohol (<14, 14–27, 28–55, ⩾56 drinks per week, plus a dummy variable for ex-drinkers) and tobacco consumption (never, ex-smoker, current smoker of <15, 15–24, ⩾25 cigarettes per day).

## RESULTS

Table 1

**Table 1:** Distribution of 965 cases of cancers of the upper aerodigestive tract and 1779 controls, and the corresponding ORs with 95% CI, according to various measures of aspirin use. Italy, 1992–2000

	**Cases**	**Controls**	**OR (95% CI)^a^**
Nonusers	928	1692	1^b^
Regular users	37	87	0.89 (0.56–1.43)
*Duration of use^c^ (years)*			
<5	29	45	1.46 (0.82–2.57)
⩾5	7	40	0.33 (0.13–0.82)
*Time since first use (years)*			
<5	23	32	1.68 (0.87–3.27)
⩾5	14	55	0.51 (0.26–0.99)

a Estimates from unconditional logistic regression adjusted for sex, age, centre, education, tobacco smoking and alcohol drinking.

b Reference category.

c The sum does not add up to the total because of some missing values.

gives the distribution of 965 upper aerodigestive cancer cases and 1779 controls according to selected measures of aspirin use. A total of 37 (3.8%) cases *vs* 87 (4.9%) controls reported regular aspirin use. Average frequency of use was 5.7 times per week among cases and 5.6 among controls; 32 cases and 78 controls took aspirin ⩾3 times per week, 27 cases and 57 controls every day. Indication of use was analgesia for 10 cases and 38 controls, whereas 27 cases and 49 controls took aspirin for cardiovascular prevention. The multivariate OR for regular use was 0.89 (95% CI 0.56–1.43). The OR decreased to 0.33 (95% CI 0.13–0.82) for users of ⩾5 years, and was 0.51 (95% CI 0.26–0.99) for ⩾5 years since first use.

A reduced risk with longer duration of aspirin use was observed for all sites considered: the ORs for ⩾5 years of use were 0.39 for oral and pharyngeal, 0.80 oesophageal and 0.09 laryngeal cancer. Similarly, the ORs for ⩾5 years since first use were 0.26, 0.66 and 0.55 for the three cancer sites, respectively.

## DISCUSSION

This study suggests that aspirin may have a beneficial effect on cancers of the upper aerodigestive tract. Although there is evidence of a possible protective effect of aspirin on oesophageal cancer (Bosetti *et al*, 2002), only scattered epidemiological data exist on its role on cancer of the oral cavity or larynx (Thun *et al*, 1993).

A significant reduced risk has been observed particularly for long-term use and in relation to a longer time since first use. These time–risk relations are similar to those described for colorectal cancer (Giovannucci *et al*, 1995, IARC, 1997; Thun *et al*, 2002), and therefore give plausibility to a causal association.

With reference to possible biological mechanisms, aspirin, as well as other nonsteroidal anti-inflammatory drugs (NSAID), acts on the arachidonic acid metabolism, blocking the synthesis of thromboxane, prostacyclin and prostaglandins, which in turn can influence cell proliferation, and hence cancer growth (Marnett, 1992; Marcus, 1995). A specific target of the protection against colorectal and other cancers by aspirin and other NSAID is the inhibition of cyclooxygenase-2, which is important for apoptosis, and therefore for control of the mechanisms of carcinogenesis (Featherstone, 1997; Hong and Sporn, 1997; Taketo, 1998a,1998b; Smith *et al*, 2000). The same mechanisms may be responsible for the favourable action of aspirin on oesophageal cancer and other cancers of the upper aerodigestive tract (Morgan and Vainio, 1998; Chan *et al*, 1999; Zimmermann *et al*, 1999; Li *et al*, 2000).

Limitations of our study should be considered that might have introduced a spurious association between aspirin use and the reduced risk of upper aerodigestive tract cancers. It is possible in fact that aspirin use has been affected by early symptoms of the conditions under study. The evidence of an association with longer use is, however, reassuring against this bias. Further, some of the diagnostic categories of the controls may be associated with increased aspirin use. However, the results were similar when cases were compared with each of the major diagnostic categories of controls, thus giving reassurance against potential selection biases. Another limitation of this study is that, although based on a large number of cases, it includes a relatively low number of regular aspirin users, reflecting the pattern of regular aspirin use in Italy. Among the strengths of the study are the similar catchment areas for cases and controls, the almost complete participation rate and the choice of hospitals controls, who are preferable to population ones with reference to reliability and validity of information on drug use, since cases and controls are similarly sensitised towards various aspects of their medical history (Kelly *et al*, 1990). Moreover, the risk estimates were adjusted for major risk factors for cancers of the upper aerodigestive cancer, that is, tobacco smoking and alcohol drinking, suggesting therefore that the inverse relation between long-term aspirin use and cancers of the upper aerodigestive tract is real.
